# Net Promoter Score: a prospective, single-centre observational study assessing if a single question determined treatment success after primary or revision hip arthroplasty

**DOI:** 10.1186/s12891-023-06981-y

**Published:** 2023-10-27

**Authors:** Katrin Osmanski-Zenk, Martin Ellenrieder, Wolfram Mittelmeier, Annett Klinder

**Affiliations:** Orthopaedische Klinik und Poliklinik, Medizinischen Fakultät, Universitaetsmedizin Rostock, Universität Rostock, Doberaner Strasse 142, D-18057 Rostock, Deutschland

**Keywords:** PROMs, Oxford Hip score, Subscales, EQ-5D, NPS

## Abstract

**Background:**

Our study aimed to identify the relationship between treatment outcome assessed by patient-reported outcomes (PROMs) and satisfaction measured by calculation of the Net Promoter Score (NPS), which identifies promoters, following total hip arthroplasty (THA). The aim was to evaluate this association separately in primary and revision THA and to determine thresholds based on PROMs that identify detractors of the surgical procedure or the centre.

**Methods:**

A total of 1,243 patients who underwent primary or revision THA at our hospital were asked to complete questionnaires of the Oxford Hip Score (OHS), Euroquol-5D (EQ-5D) and information on pain intensity preoperatively, three and 12 months after surgery. Postoperatively, the patients were additionally asked about their satisfaction with the procedure and the hospital by using three different NPS questions. The association between PROMs and NPS was evaluated based on group comparisons of primary or revision THA and receiver operating characteristics analysis (ROC) to determine threshold values.

**Results:**

At 12 months the NPS of all three questions were invariably linked to treatment outcome in patients after primary THA and patients with a single revision. In these two treatment groups, promoters always showed significantly better PROM scores than detractors. The NPS score was always higher in the primary group in comparison to the single revision group, e.g. 66.4% would undergo the procedure again in the first group, while only 33.0% would opt for this in the latter group. The high thresholds for the PROMs at 12 months, that were calculated by ROC analysis to identify promoter/detractors, indicate that patients` satisfaction required very good joint function and pain relief. However, the NPS was not a suitable tool to identify patients who need further care in an early phase after surgery.

**Conclusions:**

With NPS already a single question or a single parameter provides the desired information regarding patient satisfaction and also treatment success.

**Trial registration:**

The study was approved by the Ethics Committee at the Medical Faculty of the University Rostock: “Ethikkommission an der Medizinischen Fakultät der Universität Rostock”, Address: St.-Georg Str. 108 18055 Rostock, Germany, reference number: A2015–0055.

## Background

Quality assurance in arthroplasty depends on guideline-based patient care that aims for the best possible treatment outcome for the patient. After artificial joint replacement, main outcomes are restoring joint function, alleviating pain and as a result improving the patient’s quality of life. Whether these goals were achieved can be evaluated by clinical parameters, but also with the help of numerous, already successfully established, measuring instruments, so-called Patient-Reported Outcome Measures (PROMs) [1]. These are based on self-assessments by the patients. However, in addition to the evaluation of the treatment outcome, the evaluation of health care in terms of experiential quality is playing an increasingly important role. For this purpose, the Net Promoter Score (NPS) was developed for measuring satisfaction in economic research [2]. With a single question, e.g.: *“How likely is it that you would recommend this service to a friend and colleague?“*, so-called promoters or detractors can be identified, which either encourage or discourage others to use the respective service or product. In market research, the NPS is considered a predictor of growth [2].

While only occasionally used in Germany [3–7], internationally the NPS has already been established as a tool to assess health care quality [8]. However, the benefits in terms of measuring satisfaction with care have not yet been clearly proven [9, 10]. Based on the NPS, the government in England developed a general health care assessment and introduced in 2013 the “family and friends test (FFT)” [11]. As the FFT was criticised by both patients and healthcare professionals [11–13], it was revised so that new questions were established by the UK National Health Service (NHS) in 2020 [14].

The aim of the present study was to examine the extent to which NPS questions can be used as a measurement tool for patient satisfaction in the health care system. The influence of clinical outcome, which was measured by PROMs, on the recommendation of hip arthroplasty as assessed by NPS was to be investigated. The NPS questions were used to measure satisfaction and the recommendation rate, while the Oxford Hip Score (OHS) and the Euroquol-5D-3 L (European Quality of Life Scale − 3 level version (EQ-5D)) surveyed clinical outcome and quality of life of the patients, respectively. Our working group intended to establish a suitable tool to determine the achievement of the treatment goal of primary as well as revision hip arthroplasty postoperatively, which was to be evaluated simultaneously in accordance with patient satisfaction. Here, the focus is not only on comprehensive PROMs, but rather on a single question or measurement parameter, such as information on pain intensity or the visual analogue scale (VAS). Threshold values were determined for the PROMS as well as for the single measurement parameters, which, if falling below, indicate patient dissatisfaction.

## Methods

**Fig. 1:**
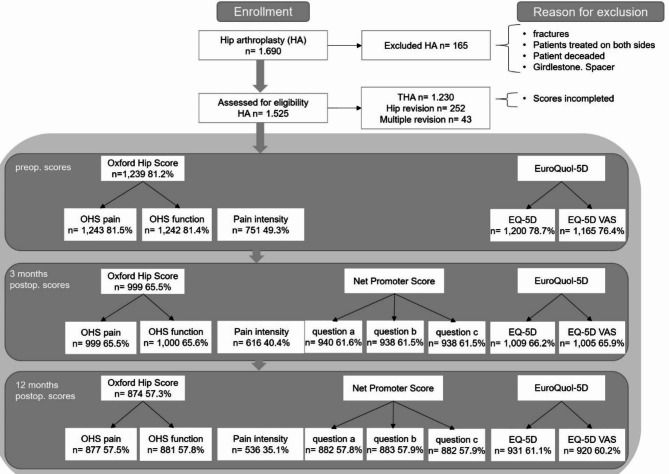
Inclusion criteria

The present follow-up study is prospective. In the period from February 2015 to February 2021, a total of 1,690 consecutive hip arthroplasty patients attended our university hospital in Germany. The patients were classified according to the surgical procedures: primary hip arthroplasty (THA, Group 1) and total hip arthroplasty revision (Groups 2 and 3). A revision was defined as the replacement or removal of at least one prosthesis component. Group 2 included patients who had a revision procedure for the first time at our institution. Group 3 included patients with more than one inpatient stay with revision surgery at our centre. Exclusion criteria are listed in Fig. 1. The surgeries were performed exclusively by experienced specialists who operate on at least 50 arthroplasties per year. The data were collected and analysed independently by the clinical research department. Ethical approval was obtained (reference number: A2015-0055).

In total, data from 1,525 patients were screened. At the time of diagnosis, the patients were informed about the study participation and consented to it accordingly. However, as not all patients completed the questionnaires, the number of patients included for data collection was lower. The number of cases who filled the respective scores and the resulting response rates are shown in Fig. 1.

### Evaluation of PROM questionnaires

For the self-assessment of the state of health, the joint-specific OHS, the health-related quality of life-related EQ-5D including the EQ-5D VAS, and information on pain intensity (0–10, 0 = no pain) were completed at the time of diagnosis as well as three and 12 months after surgery. The questionnaires were provided to the patients in paper form. At the time of diagnosis, the patients completed the questionnaires directly in our hospital, while 3 and 12 months after surgery they were sent by mail. The questionnaires were completed by the patients themselves without the involvement of third parties. The analysis of the EQ-5D was performed according to the German algorithm.

After three and 12 months, the patients were additionally asked to rate their general satisfaction with or give a recommendation of the joint surgery performed and the clinic on a five-point scale (1 yes, definitely, 2 probably yes, 3 uncertain, 4 probably not, 5 definitely not). These questions were developed in accordance with the NPS. Patients were asked if:

- in hindsight they would undergo joint operation again,
- they would recommend the joint operation they underwent to another person,
- they would recommend the centre where the joint operation was performed.

A NP score of 1 is classified as promoter (supporter), 2 as passive (undecided) and 3–5 as detractor (critic). The total score is the relative percentage of detractors subtracted from the relative percentage of promoters as a percentage. An NPS of over 50% indicates a high level of satisfaction and a good performance of the clinic [15].

Each of the three NPS questions as well as the information on pain intensity were evaluated separately. The OHS was scored according to the recommended procedure by Murray et al. [16] including the approach to missing data. For subscale analysis of “pain” and “function” the adjusted scoring scheme from 0 (worst result) to 100 (best result) by Harris et al. [17] was used.

### Statistical analysis

The statistical programme SPSS 27.0 (IBM Germany, Ehningen) was used for the statistical analysis. Statistical analysis was performed independently for the three time points and the different PROMS to ensure that the maximum number of fully completed questionnaires was evaluated. However, this resulted in a different number of patients being included in the performed comparisons (see Tables).

As Shapiro-Wilk test revealed that the data were not normally distributed, the variables were reported as median values and interquartile ranges (IQRs). Differences in PROMs by net promoter classification were assessed using the Kruskall-Wallis test with Bonferroni’s post hoc test or Mann-Whitney U test. A Chi^2^ test was performed to check the distribution of promoters, passives and detractors over both time points. Generally, p < 0.05 was considered to be statistically significant.

Receiver operating characteristic (ROC) analysis was used to determine threshold values for the respective PROMs, which predict the detractors (= anchors) if they fall below. The promoters were set as the positive value of the state variables, while the detractors were set as negative state variables. The area under the ROC curve (AUC) was used as a measure of the quality of the predictions.

## Results

While in Group 1 and Group 2 man and woman were nearly equally distributed with a slightly higher percentage of women (Group 1 53.6% and 2 54.4%), men were overrepresented in Group 3 (58.1%). The average age and body mass index (BMI) of the patient of each group was similar between the groups (Group 1: 69 years (SD 12.2), BMI 28.8 (SD 5.2), Group 2: 72 years (SD 10.8), BMI 28.1 (SD 5,7), Group 3: 71 years (SD 10,3), BMI 29.5 (SD 6.4)). However, there was a significant difference in the age between Group 1 and 2 (p = 0.008). The ASA classification showed significant differences for all three groups (p < 0.001) and ranged from 2.2 (SD 0.6) (Group 1), over 2.5 (SD 0.6) (Group 2) to 2.7 (SD 0.6) (Group 3).

The NPS of Question A was 61.3% for all patients after three months and 61.8% after one year. According to the NPS of Question B, 61.6% of patients would recommend hip arthroplasty to another person after three months and 62.2% after one year. The NPS was highest with regard to Question C, with 69.9% after three months and 69.4% after one year.

### Influence of treatment success as reported by PROM results on *NPS*

In order to assess whether the classification of patients into promoters, passives or detractors depended on the clinical outcome of the performed surgical procedure we compared the PROM scores of OHS, EQ-5D and a pain scale in these three NPS groups. While PROMs evaluate the success of the intervention from the patient’s perspective, the correlation between the selected PROM scores in this study andthe surgeon-assessed treatment success was shown by us in a previous publication [18]. As there were differences in the NPS due to the performed surgical procedure (Table 1), the comparisons were performed separately for patients who underwent primary hip arthroplasty, first revision arthroplasty or repeated revisions. The results from the comparisons are presented in Tables 2, 3, 4, 5, 6 and 7.

**Table 1:** Calculation of the NPS separately for patients with THA, single revision or multiple revisions at 3- and 12-months postoperatively

Survey time	Groups by surgical procedure	NPS question	n overall	n Promoter	Promoter in %	n Detractor	Detractor in %	NPS
3-months postoperatively	Group 1: THA	A.	816	593	72.7%	74	9.1%	**63.6%**
		B.	813	598	73.6%	62	7.6%	**65.9%**
		C.	812	635	78.2%	51	6.3%	**71.9%**
	Group 2: Hip revision	A.	106	67	63.2%	21	19.8%	**43.4%**
		B.	106	55	51.9%	21	19.8%	**32.1%**
		C.	107	72	67.3%	11	10.3%	**57.0%**
	Group 3: Multiple revision	A.	18	14	77.8%	3	16.7%	**61.1%**
		B.	19	11	57.9%	3	15.8%	**42.1%**
		C.	19	14	73.7%	3	15.8%	**57.9%**
12-months postoperatively	Group 1: THA	A.	763	575	75.4%	68	8.9%	**66.4%**
		B.	764	569	74.5%	52	6.8%	**67.7%**
		C.	764	612	80.1%	46	6.0%	**74.1%**
	Group 2: Hip revision	A.	106	63	59.4%	28	26.4%	**33.0%**
		B.	105	56	53.3%	27	25.7%	**27.6%**
		C.	104	60	57.7%	20	19.2%	**38.5%**
	Group 3: Multiple revision	A.	13	8	61.5%	5	38.5%	**23.1%**
		B.	14	8	57.1%	5	35.7%	**21.4%**
		C.	14	9	64.3%	3	21.4%	**42.9%**

A. In hindsight, would you have the joint surgery again?

B. Would you recommend the joint surgery you underwent to another person?

C. Would you recommend the clinic where your joint surgery was performed to others?

**Table 2:** Comparison of 3-months PROM scores in patients identified as promoters, passives and detractors according to NPS of Question A at 3-months postoperatively

Surgical	Name of	Number of patients identified as:			Comparison of median value of PROM score (IQR) at 3-months:			
procedure	PROM	Promoter	Passive	Detractor	Promoter	Passive	Detractor	p-value
Group 1: THA	Pain intensity	397	103	50	2 (3)^a^	4 (3)^a^	5 (4)^a^	< 0.001
	OHS	576	140	71	37 (13)^a^	30 (14)^a^	21 (13)^a^	< 0.001
	OHS pain	576	140	71	79.17 (33)^a^	58.33 (33)^a^	41.67 (29)^a^	< 0.001
	OHS function	577	140	71	75 (25)^a^	66.67 (28)^a^	50 (25)^a^	< 0.001
	EQ-5D index	547	144	71	0.900 (0.200)^a^	0.788 (0.186)^a^	0.701 (0.112)^a^	< 0.001
	EQ-5D VAS	575	143	67	80 (20)^a^	70 (30)^a^	50 (30)^a^	< 0.001
Group 2: Hip revision	Pain intensity	35	8	12	2 (3)^a^	4 (4)	6 (5)^a^	< 0.001
	OHS	61	18	21	33 (18)^a,b^	20.91 (16)^a^	18 (14)^b^	< 0.001
	OHS pain	61	18	21	70.83 (37)^a,b^	39.58 (30)^a^	33.33 (35)^b^	< 0.001
	OHS function	61	18	21	66.67 (44)^a^	37.50 (40)^a^	50 (21)	0.021
	EQ-5D index	59	17	21	0.887 (0.199)^a^	0.701 (0.087)^a^	0.701 (0.093)	0.014
	EQ-5D VAS	61	16	21	70 (35)^a^	40 (34)^a^	50 (27)	0.002
Group 3: Multiple hip revision	Pain intensity	7	1	2	5 (7)	6^#^	5 (-)	0.714
	OHS	14	1	3	26 (18)	18^#^	14.18 (-)	0.143
	OHS pain	14	1	3	56.25 (29)	33^#^	30 (-)	0.493
	OHS function	14	1	3	50 (44)^a^	42^#^	20.83 (-)^a^	0.050
	EQ-5D index	14	1	3	0.745 (0.547)	0,701^#^	0.313 (-)	0.126
	EQ-5D VAS	14	1	3	68.5 (41)	40^#^	23 (-)	0.110

Values with the same superscript letter differ significantly from each other in pairwise comparison with Bonferroni` post hoc test

Abbreviations: PROM: patient-reported outcome measure; OHS: Oxford Hip Score; VAS: visual analogue scale; IQR: interquartile range

^#^ as n = 1 the reported value represents a single value, not the median; (-): IQR values can only be calculated when n ≥ 4

After three months the response of patients in Group1 and Group 2 to NPS questions A and B was clearly linked to the treatment success, since promoters had significantly better outcomes in all the PROM scores (Tables 2 and 3). While detractors in patients with multiple revisions (Group 3) also showed worse PROM results than promoters or passives, the differences did not become significant, probably due to the small number of patients and the inter-individual differences (Tables 2, 3 and 4). Only in patients with one hospital stay (Group 1) the recommendation of the centre (Question C) after three months did depend on the treatment success as determined in all PROMs (Table 4).

**Table 3:** Comparison of 3-months PROM scores in patients identified as promoters, passives and detractors according to NPS of Question B at 3-months postoperatively

Surgical	Name of	Number of patients identified as:			Comparison of median value of PROM score (IQR) at 3-months:			
procedure	PROM	Promoter	Passive	Detractor	Promoter	Passive	Detractor	p-value
Group 1: THA	Pain intensity	396	107	46	2 (3)^a^	3 (2)^a^	5.5 (5)^a^	< 0.001
	OHS	578	148	59	36 (13)^a^	30 (15)^a^	20 (15)^a^	< 0.001
	OHS pain	578	148	59	79.17 (37)^a^	62.50 (37)^a^	41.67 (29)^a^	< 0.001
	OHS function	578	149	59	75 (29)^a^	66.67 (27)^a^	45.83 (33)	< 0.001
	EQ-5D index	577	149	60	0.887 (0.200)^a^	0.887 (0.211)^a^	0.701 (0.459)^a^	< 0.001
	EQ-5D VAS	578	148	57	80 (20)^a^	70 (30)^a^	60 (35)^a^	< 0.001
Group 2: Hip revision	Pain intensity	28	11	16	2 (2)^a^	3 (4)^b^	6.5 (5)^a,b^	< 0.001
	OHS	50	29	21	35 (18)^a,b^	22 (15)^a^	18 (8)^b^	< 0.001
	OHS pain	50	29	21	75 (34)^a,b^	45.83 (42)^a^	33.33 (21)^b^	< 0.001
	OHS function	50	29	21	68.75 (42)^a^	50 (29)	37.50 (29)^a^	0.001
	EQ-5D index	48	28	21	0.887 (0.276)^a^	0.788 (0.167)	0.701 (0.323)^a^	0.004
	EQ-5D VAS	49	28	21	70 (38)^a,b^	55 (30)^a^	50 (27)^b^	0.004
Group 3: Multiple hip revision	Pain intensity	5	3	2	5 (6)	0 (-)	5 (-)	0.442
	OHS	11	5	3	22 (19)	31 (16)	14.18 (-)	0.241
	OHS pain	11	5	3	50 (25)	75 (31)	30 (-)	0.138
	OHS function	11	5	3	41.67 (50)	54.17 (46)	29.17 (-)	0.344
	EQ-5D index	11	5	3	0.701 (0.410)	0.701 (0.486)	0.313 (-)	0.150
	EQ-5D VAS	11	5	3	70 (45)	40 (45)	40 (-)	0.156

Values with the same superscript letter differ significantly from each other in pairwise comparison with Bonferroni` post hoc test

Abbreviations: PROM: patient-reported outcome measure; OHS: Oxford Hip Score; VAS: visual analogue scale; IQR: interquartile range

^#^ as n = 1 the reported value represents a single value, not the median; (-): IQR values can only be calculated when n ≥ 4

**Table 4:** Comparison of 3-months PROM scores in patients identified as promoters, passives and detractors according to NPS of Question C at 3-months postoperatively

Surgical	Name of	Number of patients identified as:			Comparison of median value of PROM score (IQR) at 3-months:			
procedure	PROM	Promoter	Passive	Detractor	Promoter	Passive	Detractor	p-value
Group 1: THA	Pain intensity	418	96	33	2 (4)^a^	3 (3)^a^	5 (5)^a^	< 0.001
	OHS	613	124	47	36 (14)^a^	31 (17)^a^	19 (16)^a^	< 0.001
	OHS pain	613	124	47	75 (33)^a,b^	62.50 (42)^a^	41.67 (33)^b^	< 0.001
	OHS function	613	124	48	75 (29)^a,b^	66.67 (29)^a^	45.83 (37)^b^	< 0.001
	EQ-5D index	615	121	49	0.887 (0.212)^a^	0.800 (0.186)^a^	0.701 (0.542)^a^	< 0.001
	EQ-5D VAS	611	124	47	80 (20)^a,b^	70 (30)^a^	65 (40)^b^	< 0.001
Group 2: Hip revision	Pain intensity	38	13	4	2 (3)^a^	5 (4)	7 (4)^a^	0.003
	OHS	66	24	11	30.50 (20)^a^	20.50 (18)	18 (9)^a^	0.025
	OHS pain	66	24	11	68.75 (42)^a^	43.75 (44)	37.50 (29)^a^	0.006
	OHS function	66	24	11	64.58 (43)	45.83 (32)	50 (37)	0.136
	EQ-5D index	67	20	11	0.800 (0.186)	0.701 (0.109)	0.701 (0.509)	0.129
	EQ-5D VAS	65	23	11	64 (43)	60 (20)	50 (40)	0.424
Group 3: Multiple hip revision	Pain intensity	7	1	2	5 (7)	0^#^	5 (-)	0.441
	OHS	14	2	3	23.50 (19)	27.50 (-)	14.18 (-)	0.297
	OHS pain	14	2	3	52.08 (32)	77.08 (-)	30 (-)	0.179
	OHS function	14	2	3	43.75 (44)	37.50 (-)	29.17 (-)	0.259
	EQ-5D index	14	2	3	0.745 (0.547)	0.464 (-)	0.313 (-)	0.087
	EQ-5D VAS	14	2	3	65 (41)	45 (-)	40 (-)	0.240

Values with the same superscript letter differ significantly from each other in pairwise comparison with Bonferroni` post hoc test

Abbreviations: PROM: patient-reported outcome measure; OHS: Oxford Hip Score; VAS: visual analogue scale; IQR: interquartile range

^#^ as n = 1 the reported value represents a single value, not the median; (-): IQR values can only be calculated when n ≥ 4

This difference between Questions A and B compared to Question C at three months was no longer apparent at 12 months postoperatively. At 12 months the NPS of all three questions were invariably linked to treatment outcome in Groups 1 and 2, since promoters always showed significantly better PROM scores than detractors in both groups (Tables 5, 6 and 7). At 12 months, promoters in Group 3 also displayed mostly better PROM scores than detractors, however, these differences were only significant for very few PROM scores (Tables 5 and 6: Pain intensity at 12 months), while a slightly higher number showed a statistical trend (0.05 < p < 0.1), e.g. OHS function at 12 months postoperatively for Questions A, B and C (Tables 5, 6 and 7). At 12 months postoperatively patients with multiple revisions (Group 3) had formed a firm opinion of whether they would undergo the procedure again or would recommend it to another person (Questions A and B) as they were either promoters or detractors. None of the patients in Group 3 were undecided (passives) at 12 months with regard to Question A and hardly any with regard to Question B (Tables 6 and 7).

**Table 5:** Comparison of 3-months and 12-months PROM scores in patients identified as promoters, passives and detractors according to NPS of Question A at 12-months postoperatively

Surgical	Name of	Survey time	Number of patients identified as:			Comparison of median value of PROM score (IQR) at 12-months:			
procedure	PROM		Promoter	Passive	Detractor	Promoter	Passive	Detractor	p-value
Group 1: THA	Pain intensity	3-months	340	72	40	2 (3)^a,b^	3 (3)^a^	4 (3)^b^	< 0.001
	OHS		512	102	57	37 (13)^a,b^	30.50 (15)^a^	25 (18)^b^	< 0.001
	OHS pain		512	102	57	79.17 (33)^a^	62.50 (33)^a^	54.17 (40)^a^	< 0.001
	OHS function		512	103	57	77.08 (25)^a,b^	66.67 (29)^a^	58.33 (33)^b^	< 0.001
	EQ-5D index		503	99	54	0.887 (0.113)^a,b^	0.788 (0.186)^a^	0.788 (0.186)^b^	< 0.001
	EQ-5D VAS		497	100	53	80 (20)^a,b^	70 (29)^a^	60 (25)^b^	< 0.001
	Pain intensity	12-months	331	73	54	1 (3)^a,b^	3 (3)^a^	5 (3)^b^	< 0.001
	OHS		567	118	67	44 (9)^a,b^	38 (13)^a^	29 (18)^b^	< 0.001
	OHS pain		568	119	67	91.67 (21)^a^	75 (29)^a^	54.17 (46)^a^	< 0.001
	OHS function		571	118	68	91.67 (21)^a,b^	79.17 (25)^a^	66.67 (37)^b^	< 0.001
	EQ-5D index		558	116	64	1.000 (0.113)^a^	0.887 (0.189)^a^	0.788 (0.459)^a^	< 0.001
	EQ-5D VAS		548	114	65	85.50 (24)^a,b^	70 (35)^a^	60 (35)^b^	< 0.001
Group 2: Hip revision	Pain intensity	3-months	25	4	16	2 (3)^a^	3 (2)	5.5 (4)^a^	0.002
	OHS		50	12	21	33.50 (16)^a^	26.50 (9)	18 (16)^a^	< 0.001
	OHS pain		50	12	21	70.83 (37)^a^	56.25 (17)	37.50 (40)^a^	< 0.001
	OHS function		50	12	21	66.67 (43)^a^	58.33 (16)	37.50 (29)^a^	0.002
	EQ-5D index		49	10	21	0.887 (0.186)^a^	0.745 (0.186)	0.701 (0.323)^a^	< 0.001
	EQ-5D VAS		47	10	21	70 (35)^a,b^	50 (23)^a^	50 (25)^b^	0.002
	Pain intensity	12-months	33	12	19	1 (5)^a^	3 (3)	7 (5)^a^	0.002
	OHS		63	15	28	39 (21)^a^	33 (18)^b^	19.50 (22)^a,b^	< 0.001
	OHS pain		63	15	28	79.17 (37)^a^	70.83 (33)	37.08 (52)^a^	< 0.001
	OHS function		63	15	28	79.17 (37)^a^	70.83 (35)^b^	45.83 (37)^a,b^	< 0.001
	EQ-5D index		61	14	27	0.887 (0.299)^a^	0.801 (0.189)	0.701 (0.613)^a^	< 0.001
	EQ-5D VAS		61	15	27	75 (40)^a^	60 (30)	40 (50)^a^	0.002
Group 3: Multiple hip revision	Pain intensity	3-months	3	0	4	5 (-)	n/a	5 (9)	1,000
	OHS		8	0	5	19.50 (21)	n/a	22 (22)	0.943
	OHS pain		8	0	5	50 (44)	n/a	50 (66)	0.724
	OHS function		8	0	5	35.42 (49)	n/a	29.17 (54)	0.524
	EQ-5D index		8	0	5	0.745 (0.569)	n/a	0.313 (0.489)	0.127
	EQ-5D VAS		8	0	5	70 (38)	n/a	40 (58)	0.354
	Pain intensity	12-months	7	0	5	4 (5)	n/a	7 (3)	0.048
	OHS		8	0	5	20.20 (32)	n/a	15 (8)	0.127
	OHS pain		8	0	5	43.33 (74)	n/a	25 (21)	0.622
	OHS function		8	0	5	41.67 (55)	n/a	25 (21)	0.093
	EQ-5D index		7	0	5	0.701 (0.299)	n/a	0.701 (0.339)	0.149
	EQ-5D VAS		7	0	4	60 (50)	n/a	40 (60)	0.527

Values with the same superscript letter differ significantly from each other in pairwise comparison with Bonferroni` post hoc test

Abbreviations: PROM: patient-reported outcome measure; OHS: Oxford Hip Score; VAS: visual analogue scale; IQR: interquartile range

n/a: not applicable as n = 0

**Table 6:** Comparison of 3-months and 12-months PROM scores in patients identified as promoters, passives and detractors according to NPS of Question B at 12-months postoperatively

Surgical	Name of	Survey time	Number of patients identified as:			Comparison of median value of PROM score (IQR) at 12-months:			
procedure	PROM		Promoter	Passive	Detractor	Promoter	Passive	Detractor	p-value
Group 1: THA	Pain intensity	3-months	337	86	31	2 (3)^a^	3 (3)^a^	5 (2)^a^	< 0.001
	OHS		501	127	43	37 (12)^a^	31 (15)^a^	25 (13)^a^	< 0.001
	OHS pain		501	127	43	79.17 (33)^a^	62.50 (33)^a^	50 (33)^a^	< 0.001
	OHS function		501	128	43	79.17 (25)^a,b^	66.67 (29)^a^	58.33 (29)^b^	< 0.001
	EQ-5D index		494	121	42	0.887 (0.131)^a^	0.800 (0.112)^a^	0.701 (0.090)^a^	< 0.001
	EQ-5D VAS		491	120	40	80 (20)^a,b^	70 (30)^a^	60 (24)^b^	< 0.001
	Pain intensity	12-months	332	88	40	1 (3)^a^	3 (3)^a^	5 (3)^a^	< 0.001
	OHS		560	141	51	44 (9)^a^	38 (15)^a^	26 (19)^a^	< 0.001
	OHS pain		560	143	51	91.67 (21)^a^	75 (33)^a^	45.83 (42)^a^	< 0.001
	OHS function		565	141	52	91.67 (21)^a^	79.17 (25)^a^	60.42 (30)^a^	< 0.001
	EQ-5D index		550	139	50	1.000 (0.113)^a^	0.887 (0.212)^a^	0.788 (0.491)^a^	< 0.001
	EQ-5D VAS		544	134	50	85 (25)^a^	70 (25)^a^	50 (41)^a^	< 0.001
Group 2: Hip revision	Pain intensity	3-months	20	9	16	2 (2)^a^	3 (4)	6 (3)^a^	< 0.001
	OHS		45	15	22	36 (15)^a,b^	22 (11)^a^	16.50 (12)^b^	< 0.001
	OHS pain		45	15	22	75 (29)^a,b^	45.83 (21)^a^	35.42 (30)^b^	< 0.001
	OHS function		45	15	22	70.83 (31)^a,b^	50 (25)^a^	37.50 (19)^b^	< 0.001
	EQ-5D index		43	14	22	0.887 (0.112)^a^	0.701 (0.267)	0.701 (0.339)^a^	< 0.001
	EQ-5D VAS		41	14	22	70 (35)^a^	52.50 (33)	50 (31)^a^	0.001
	Pain intensity	12-months	28	15	20	1 (4)^a,b^	4 (4)^a^	7 (4)^b^	< 0.001
	OHS		56	22	27	41 (17)^a,b^	30 (19)^a^	18 (21)^b^	< 0.001
	OHS pain		56	22	27	87.50 (33)^a,b^	68.75 (45)^a^	29.17 (50)^b^	< 0.001
	OHS function		56	22	27	83.33 (33)^a,b^	64.58 (51)^a^	45.83 (37)^b^	< 0.001
	EQ-5D index		54	21	26	0.894 (0.234)^a^	0.788 (0.598)	0.701 (0.629)^a^	< 0.001
	EQ-5D VAS		54	22	26	75 (30)^a,b^	57.50 (41)^a^	37.50 (50)^b^	< 0.001
Group 3: Multiple hip revision	Pain intensity	3-months	3	0	4	5 (-)	n/a	5 (9)	1,000
	OHS		8	1	5	19.50 (21)	26^#^	22 (22)	0.816
	OHS pain		8	1	5	50 (44)	67^#^	50 (66)	0.877
	OHS function		8	1	5	35.42 (49)	42^#^	29.17 (54)	0.683
	EQ-5D index		8	1	5	0.745 (0.569)	0,175^#^	0.313 (0.489)	0.106
	EQ-5D VAS		8	1	5	70 (38)	40^#^	40 (58)	0.451
	Pain intensity	12-months	7	0	5	4 (5)	n/a	7 (3)	0.047
	OHS		8	0	5	20.20 (32)	n/a	15 (8)	0.123
	OHS pain		8	0	5	43.33 (74)	n/a	25 (21)	0.555
	OHS function		8	0	5	41.67 (55)	n/a	25 (21)	0.075
	EQ-5D index		7	0	5	0.701 (0.299)	n/a	0.701 (0.339)	0.064
	EQ-5D VAS		7	1	4	60 (50)	30^#^	40 (60)	0.371

Values with the same superscript letter differ significantly from each other in pairwise comparison with Bonferroni` post hoc test

Abbreviations: PROM: patient-reported outcome measure; OHS: Oxford Hip Score; VAS: visual analogue scale; IQR: interquartile range

^#^ as n = 1 the reported value represents a single value, not the median; n/a: not applicable as n = 0; (-): IQR values can only be calculated when n ≥ 4

There were no significant differences between promoters, passives or detractors when analysing the pre-operative PROM scores. This supports the notion that the identification of patients as promoters, passives or detractors according to Questions A, B and C is clearly outcome-related.

### Comparison of the distribution of promoters, passives and *detractors*

**Fig. 2:**
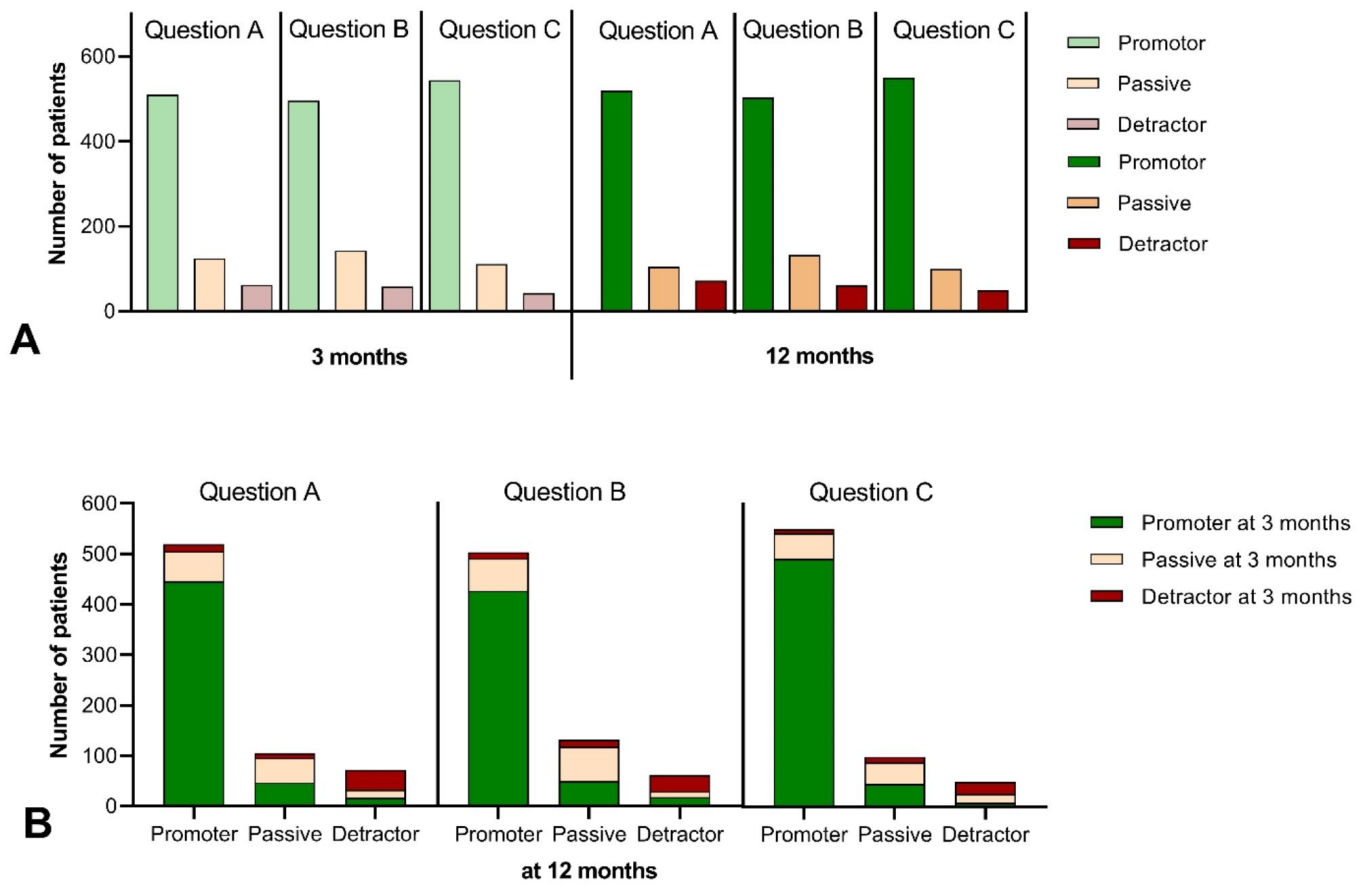
Distribution of promoters, passives and detractors over time. (**A**) Comparison of the total number of promoters, passives and detractors between 3- and 12-months. (**B**) Illustration of the change of opinion in patients at 12-months in relation to their NPS status at 3-months

As patient`s circumstance can change over time, for instance by further improvement of joint function but also deterioration of general health, we studied changes in the distribution of promoters, passives and detractors over both time points. Only patients that responded to the NPS questions at both time points were included in the analysis. The comparison of three and 12 months showed an overall increase in promoters at 12 months (Fig. 2A).

**Table 7:** Comparison of 3-months and 12-months PROM scores in patients identified as promoters, passives and detractors according to NPS of Question C at 12-months postoperatively

Surgical	Name of	Survey time	Number of patients identified as:			Comparison of median value of PROM score (IQR) at 12-months:			
procedure	PROM		Promoter	Passive	Detractor	Promoter	Passive	Detractor	p-value
Group 1: THA	Pain intensity	3-months	362	64	27	2 (3)^a^	3 (2)^a^	5 (3)^a^	< 0.001
	OHS		544	93	34	37 (14)^a,b^	32 (14)^a^	26.50 (14)^b^	< 0.001
	OHS pain		544	93	34	79.17 (37)^a,b^	62.50 (33)^a^	54.17 (39)^b^	< 0.001
	OHS function		544	93	35	75 (29)^a,b^	66.67 (21)^a^	62.50 (29)^b^	< 0.001
	EQ-5D index		536	88	33	0.887 (0.200)^a,b^	0.807 (0.186)^a^	0.788 (0.186)^b^	< 0.001
	EQ-5D VAS		527	91	33	80 (20)^a,b^	70 (30)^a^	70 (35)^b^	< 0.001
	Pain intensity	12-months	353	71	35	1 (3)^a,b^	3 (3)^a^	5 (4)^b^	< 0.001
	OHS		601	105	46	43 (9)^a,b^	37 (15)^a^	29.50 (20)^b^	< 0.001
	OHS pain		603	105	46	91.67 (25)^a,b^	75 (33)^a^	52.08 (48)^b^	< 0.001
	OHS function		607	105	46	91.67 (21)^a,b^	79.17 (25)^a^	66.67 (43)^b^	< 0.001
	EQ-5D index		593	101	45	1.000 (0.113)^a^	0.887 (0.212)^a^	0.788 (0.186)^a^	< 0.001
	EQ-5D VAS		588	97	43	85 (25)^a,b^	75 (25)^a^	60 (40)^b^	< 0.001
Group 2: Hip revision	Pain intensity	3-months	27	8	10	2 (2)^a,b^	5 (4)^a^	6 (2)^b^	< 0.001
	OHS		50	17	15	32.50 (17)^a^	23 (15)	18 (9)^a^	0.002
	OHS pain		50	17	15	70.83 (37)^a^	54.17 (31)	37.50 (33)^a^	0.002
	OHS function		50	17	15	66.67 (46)^a^	50 (27)	37.50 (25)^a^	0.008
	EQ-5D index		48	16	15	0.877 (0.196)^a^	0.745 (0.109)	0.701 (0.323)^a^	0.001
	EQ-5D VAS		46	16	15	65.50 (45)	60 (20)	50 (35)	0.151
	Pain intensity	12-months	31	18	14	3 (5)^a^	35 (6)^b^	7.5 (5)^a,b^	< 0.001
	OHS		60	24	20	39 (17)^a^	32.70 (20)^b^	18 (19)^a,b^	< 0.001
	OHS pain		60	24	20	81.25 (41)^a^	66.67 (35)^b^	29.17 (41)^a,b^	< 0.001
	OHS function		60	24	20	79.17 (36)^a^	70.83 (46)^b^	43.75 (35)^a,b^	< 0.001
	EQ-5D index		58	22	20	0.887 (0.299)^a^	0.788 (0.824)	0.482 (0.591)^a^	< 0.001
	EQ-5D VAS		59	23	19	80 (40)^a,b^	60 (40)^a^	40 (50)^b^	< 0.001
Group 3: Multiple hip revision	Pain intensity	3-months	4	1	2	5 (5)	0^#^	5 (-)	0.293
	OHS		9	2	3	20 (21)	24 (-)	14.18 (-)	0.315
	OHS pain		9	2	3	54.17 (44)	58.33 (-)	30 (-)	0.822
	OHS function		9	2	3	37.50 (52)	41.67 (0)	20.83 (-)	0.074
	EQ-5D index		9	2	3	0.788 (0.622)^a^	0.175 (0)^a^	0.313 (-)	0.018
	EQ-5D VAS		9	2	3	80 (40)	40 (0)	23 (-)	0.094
	Pain intensity	12-months	8	1	3	5 (5)	7^#^	7 (-)	0.527
	OHS		9	1	3	20 (31)	19^#^	11 (-)	0.238
	OHS pain		9	1	3	41.67 (73)	33^#^	20.83 (-)	0.875
	OHS function		9	1	3	41.67 (57)	46^#^	20.83 (-)	0.075
	EQ-5D index		8	1	3	0.701 (0.644)	0,701^#^	0.701 (-)	0.202
	EQ-5D VAS		8	2	2	55 (45)	25 (-)	70 (-)	0.107

Values with the same superscript letter differ significantly from each other in pairwise comparison with Bonferroni` post hoc test

Abbreviations: PROM: patient-reported outcome measure; OHS: Oxford Hip Score; VAS: visual analogue scale; IQR: interquartile range

^#^ as n = 1 the reported value represents a single value, not the median; (-): IQR values can only be calculated when n ≥ 4

However, the dynamic of these changes was more complex (Fig. 2B). For example, with Question A a total of 510 patients were identified as promoters at three months. Of these, 9.2% became passives and 3.3% became detractors at 12 months. At the same time, 62 patients were detractors at three months, of whom more than 22.6% were recovered as promoters and 14.5% as passives at 12 months. Finally, 62.9% of this group still remained as detractors after 12 months. Questions B and C also showed similar trends (Fig. 2B).

### ROC-Analysis

**Fig. 3:**
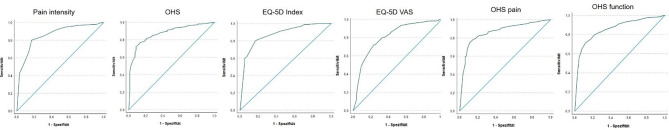
ROC-curve Question B 12-months postoperatively

The ROC analyses allowed the calculation of thresholds in the PROM scores that indicate whether patients identify as detractors for the respective NPS questions (Table 8; Fig. 3). Based on our ROC analyses, we can recommend all the measurement instruments examined, such as the OHS, including both subscores, the EQ-5D and also the pain intensity measure for predicting patient satisfaction after hip replacement. All scores show good (AUC > 0.7) to very good model qualities (AUC > 0.8) both after three and after 12 months [19].

**Table 8:** ROC analyses of NPS questions A, B and C at 3- and 12-months postoperatively

**Question A**	PROM/ Measurement	AUC (95% CI)	p-value	Threshold value	Model quality
3-months postoperative	Pain intensity	0.811 (0.753–0.870)	< 0.001	3.5	0.75
	OHS	0.797 (0.752–0.842)	< 0.001	31.32	0.75
	OHS pain	0.773 (0.726–0.821)	< 0.001	61.25	0.73
	OHS function	0.780 (0.734–0.826)	< 0.001	65.83	0.73
	EQ-5D index	0.823 (0.780–0.865)	< 0.001	0.794	0.78
	EQ-5D VAS	0.817 (0.775–0.859)	< 0.001	72.5	0.78
12-months postoperative	Pain intensity	0.806 (0.751–0.861)	< 0.001	3.5	0.75
	OHS	0.835 (0.796–0.874)	< 0.001	38.50	0.80
	OHS pain	0.830 (0.790–0.870)	< 0.001	77.08	0.79
	OHS function	0.810 (0.766–0.854)	< 0.001	84.17	0.77
	EQ-5D index	0.860 (0.820–0.900)	< 0.001	0.794	0.82
	EQ-5D VAS	0.784 (0.733–0.835)	< 0.001	77.50	0.73
**Question B**	PROM/ Measurement	AUC (95% CI)	p-value	Threshold value	Model quality
3-months postoperative	Pain intensity	0.849 (0.801–0.896)	< 0.001	2.5	0.80
	OHS	0.806 (0.755–0.857)	< 0.001	26.09	0.75
	OHS pain	0.788 (0.738–0.838)	< 0.001	56.25	0.74
	OHS function	0.786 (0.732–0.841)	< 0.001	54.58	0.73
	EQ-5D index	0.820 (0.771–0.869)	< 0.001	0.8065	0.77
	EQ-5D VAS	0.775 (0722-0.827)	< 0.001	64.5	0.72
12-months postoperative	Pain intensity	0.859 (0.811–0.906)	< 0.001	3.5	0.81
	OHS	0.873 (0.838–0.908)	< 0.001	38.50	0.84
	OHS pain	0.863 (0.824–0.903)	< 0.001	77.08	0.82
	OHS function	0.857 (0.818–0.895)	< 0.001	81.25	0.82
	EQ-5D index	0.872 (0.831–0.912)	< 0.001	0.8065	0.83
	EQ-5D VAS	0.808 (0.755–0.860)	< 0.001	73.50	0.75
**Question C**	PROM/ Measurement	AUC (95% CI)	p-value	Threshold value	Model quality
3-months postoperative	Pain intensity	0.794 (0.722–0.867)	< 0.001	3.5	0.72
	OHS	0.801 (0.747–0.855)	< 0.001	26.09	0.75
	OHS pain	0.774 (0.717–0.831)	< 0.001	59.17	0.72
	OHS function	0.776 (0.718–0.834)	< 0.001	68.75	0.72
	EQ-5D index	0.789 (0.729–0.848)	< 0.001	0.7445	0.73
	EQ-5D VAS	0.727 (0.662–0.793)	< 0.001	72.50	0.66
12-months postoperative	Pain intensity	0.821 (0.765–0.876)	< 0.001	3.5	0.77
	OHS	0.805 (0.750–0.860)	< 0.001	36.50	0.75
	OHS pain	0.805 (0.747–0.863)	< 0.001	77.08	0.75
	OHS function	0.778 (0.717–0.838)	< 0.001	77.08	0.72
	EQ-5D index	0.815 (0.762–0.869)	< 0.001	0.794	0.76
	EQ-5D VAS	0.754 (0.687–0.822)	< 0.001	67.50	0.69

After 12 months, the thresholds of all PROM scores apart from EQ-5D VAS at Question C were higher compared to those at three months. Interestingly, for pain intensity, where lower values indicate an improvement, threshold values either remained stable (Questions A and C) or increased (Question B) from three to 12 months.

## Discussion

Our study results showed that after 12 months, the NPS questions used here were associated with treatment outcome after primary THA care (Group 1) as well as after a single revision (Group 2). In both treatment groups, promoters showed significantly better PROM scores than detractors. To complement this, we determined threshold values for the PROMs studied here that predict the so-called promoters, consequently satisfied patients with very good joint function and pain relief.

While the use of the NPS as a benchmarking system for customer satisfaction is widespread in the market economy, this score is hardly used in the German health care system. In England, on the other hand, the NPS is an unambiguous signpost for patients [20]. The NPS of hospitals are mostly between 65 and 80 [21]. Satisfaction rates of primary hip arthroplasty are generally high with approximately 90% of patients reporting to be satisfied with their operation [22–24]. NPS was previously used in two studies to specifically asses patient satisfaction in primary THA, but not in revision. The studies of Hamilton et al. [15] and Lynskey et al. [25] used questions similar to Question B – *“Would you recommend this operation to someone else?”* or *“how likely is it that you would recommend this procedure to a friend or family?“*, respectively – to calculate the NPS. Compared to their NPS results of 71 and 79 after 1 year or 1–5 years, respectively, the NPS of Question B in Group 1 was slightly lower in our centre at three months, but improved to a similar value at 12 months. To our knowledge, however, this is the first study to use NPS to assess patient satisfaction after revision arthroplasty of the hip. The NPS of the revision patients were still favourable to excellent, but the values were markedly lower and, contrary to primary THA, values decreased over time for all three questions. This is in accordance with previous reports that showed that functional outcome, quality of life, but also satisfaction were lower after revision THA compared to primary THA [26–28]. Eisler et al. [26] reported with 63% only moderate satisfaction after revision THA compared to the 90% recorded in studies of primary THA [22–24]. Similar results were also observed by Lübbecke et al. [27] in a direct comparison five years after revision and primary THA with satisfaction rates of 67% and 84%, respectively. Several of these studies reported a correlation between satisfaction rating and disease-specific PROMs [23, 26, 29]. Therefore, we also analysed PROM results in relation to NPS classification.

When relating the satisfaction of patients to the preoperative, 3-months and 12-months PROM results, the observation by Judge et al. [30] that preoperative OHS scores did not allow the prediction of satisfied or dissatisfied patients, was confirmed by the results from our study. There were no differences in preoperative PROM scores according to NPS classification. For the postoperative PROM scores, it was more apparent at 12 months than at three months, that the identification of patients as promoters, passives and detractors depended on clinical outcome and quality of life. The relationship between NPS classification and success of intervention was in accordance to Hamilton et al. [15] who reported that a model of four variables including the satisfaction with pain relief, meeting of expectations, and the hospital experience predicted 95% of NPS responses. Similarly, Anakwe et al. [31] found the highest correlations to overall satisfaction for the individual factors “relief of pain” and “meeting patient expectation”, closely followed by “increased activity” as a functional outcome. The importance of pain relief for patient satisfaction was also highlighted by the study of Halawi et al. [32]. This is interesting with regard to our study, since in patients with a single revision, classification according to NPS of Question C after three months was related to both pain scores but not to the functional outcome. Even in Group 3 after 12 months, pain intensity was the only measure that was significantly different between promoters and detractors, thus highlighting relief of pain as the most sensitive factor for patient satisfaction, in particular in revision patients.

Over time, more patients became promoters, but also the number of detractors increased. Consequently, only the number of passives declined over both measurement time points, indicating that patients` opinion regarding their surgery consolidated from three to 12 months. This is reflected by the ROC calculated thresholds. Apart from pain intensity all other outcome measures showed higher thresholds, i.e. a demand for more beneficial outcomes, at 12 months compared to three months. This suggests increased expectations at 12 months after surgery, probably also due to improved functional outcome and quality of life.

In order to simplify the assessment of individual treatment success, it has been extensively discussed whether a single question, for example a NPS question, might be sufficient for evaluating the outcome after surgery. While some researchers promoted the idea of a single question [33], other were more critical as they saw no additional benefit in the use of the NPS with regard to patient experience [10]. When comparing the thresholds to identify patients who require further treatment after three month (34.50, 72.92 and 0.794 for OHS, OHS functional score and EQ-5D, respectively [18]) to the 3-months thresholds for identifying detractors, the latter are markedly lower. Therefore, at three months, patients were promoters even though they had comparatively poor scores, might not have reached the treatment goal and required further intervention. Consequently, the NPS is not a suitable tool to identify patients who need further care in an early phase after surgery. However, over time the thresholds, that separate promoters from detractors, increased and for example the value of 38.5 for OHS after 12 months for Questions A and B was the same as reported for satisfied patients after 12 month by Kjærgaard et al. [34] and similar to values reported for follow-up periods of 6 months or three years [35, 36]. The high thresholds of the present study at 12 months make it obvious that patients` satisfaction does require very good joint function and pain relief. The improvements over time including the higher expectations might also be based on our follow-up care algorithm that takes in account several patient factors [18, 37]. Hamilton et al. [38], who calculated ROC thresholds for “treatment success” based on OHS after 12 months, also suggested that a composite anchor criterion comprising patient satisfaction, functional improvement, pain relief, and willingness to undergo the same procedure again allows for a more detailed assessment of the individual patients. The number of patients in their study who reported a “successful outcome” due to the combined criterion was notably lower than the number of patients who reported being “satisfied”. Thus, more patients without a successful outcome can be identified by evaluating a combination of factors, instead of only asking for patients` satisfaction. Threshold values also varied when patients were grouped according to their preoperative PROM scores. This suggests that pain and physical restrictions due to comorbidities may influence any outcome score and NPS. Ellenrieder et al. [39] showed that coexistent lumbar spine disorders have a crucial impact on the clinical outcome (Short-Form-36) after total hip replacement. However, factors such as older age, lower preoperative function, and a higher number of medical or orthopaedic comorbidities impacted on outcome measures mainly after primary THA, while in revision THA these factors were only partly responsible for the worse outcome after revision surgery [27].

When comparing the three different NPS questions, it was Question C that always scored the highest, independent from the follow-up period or the treatment group. It is likely, that additional factors to the here studied pain relief, function and quality of life had a greater impact on the recommendation of the centre. Hamilton et al. [15] identified “hospital experience” as one of the crucial factors in NPS response even for the recommendation of the surgery to others. Hospital loyalty was strongly associated with waiting time experience, nursing care experience and the experienced communication with the physician [40]. The influence of these additional factors might make Question C less suitable in assessing treatment success.

### Strengths and limitations

Our study allowed the assessment of patient satisfaction after THA over two different follow-up periods, an early time point of three months, when an intervention such as physical therapy might still be initiated to improve functional outcome, and a later time point of 12 months, when it is thought that recovery after surgery is complete. To our knowledge this is also the first study to use NPS to evaluate patient satisfaction in primary as well as in revision THA. While the further subdivision of revision THA in single and multiple revisions resulted in a low number of patients in Group 3 and thus limited the validity of the results for this group, we decided that the often very poor clinical outcome in this last group of patients necessitated subdivision. However it has to be noted that, when defining Group 2 and 3, it was not enquired whether any of these patients had already had one or more revision surgeries in other clinics.

As referred to in the discussion, hospital experience is an important factor for NPS response. Thus, a limitation regarding the interpretation of our results is that we did not collect any data about hospital experience. We also did not consider comorbidities or psychosocial or geriatric aspects.

## Conclusion

NPS represent an easy-to-use tool to measure patients` satisfaction and thus treatment success after primary and revision hip arthroplasty. Their use considerably minimises the survey effort, both for the patients and for the medical institution. However, they lack the differentiated assessment provided by PROM scores that allows the identification of patients who require further care, especially early after surgery. Questions regarding recommendation to another person or to undergo the operation again are more suitable in assessing treatment success than recommendation of the centre. Pain relief was the most sensitive factor for patient satisfaction, in particular in revision patients.

### Outlook

Various factors affect the PROM values three and 12 months postoperatively, but these are not necessarily reflected in patient satisfaction. Therefore, we want to examine the influence of comorbidities, gender, age, BMI, but also complications during the early postoperative phase until discharge on the NPS and the threshold values in further studies.

## Data Availability

The datasets analysed during the current study are not publicly available due to the missing consent from all patients but are available in excerpts from the corresponding author on reasonable request.

## Abbreviations

AUC: area under the ROC curve
BMI: body mass index
EQ-5D: European Quality of Life Scale − 3 level version
FFT: family and friends test
IQRs: interquartile ranges
NHS: National Health Service
NPS: Net Promoter Score
OHS: Oxford Hip Score
PROMs: patient-reported outcomes
ROC: Receiver operating characteristic
THA: total hip arthroplasty
VAS: visual analogue scale
